# Cobalt–Iron–Phosphate Hydrogen Evolution Reaction Electrocatalyst for Solar-Driven Alkaline Seawater Electrolyzer

**DOI:** 10.3390/nano11112989

**Published:** 2021-11-06

**Authors:** Chiho Kim, Seunghun Lee, Seong Hyun Kim, Jaehan Park, Shinho Kim, Se-Hun Kwon, Jong-Seong Bae, Yoo Sei Park, Yangdo Kim

**Affiliations:** 1 Department of Materials Science and Engineering, Pusan National University, Busan 46241, Korea; chihokim@pusan.ac.kr (C.K.); basicroof@hanmail.net (S.L.); kshe1995@naver.com (S.H.K.); parkoo68@pusan.ac.kr (J.P.); sehun@pusan.ac.kr (S.-H.K.); 2 BK21 Four, Innovative Graduate Education Program for Global High-Tech Materials & Parts, Pusan National University, Busan 46241, Korea; shinho@pusan.ac.kr; 3 Busan Center, Korea Basic Science Institute, Busan 46724, Korea; jsbae@kbsi.re.kr; 4 Department of Chemical Engineering, Kansas State University, 1701A Platt St., Manhattan, KS 66506, USA

**Keywords:** seawater splitting, hydrogen evolution reaction, cobalt-iron-phosphate electrocatalysts, phosphidation, hydrogen energy

## Abstract

Seawater splitting represents an inexpensive and attractive route for producing hydrogen, which does not require a desalination process. Highly active and durable electrocatalysts are required to sustain seawater splitting. Herein we report the phosphidation-based synthesis of a cobalt–iron–phosphate ((Co,Fe)PO_4_) electrocatalyst for hydrogen evolution reaction (HER) toward alkaline seawater splitting. (Co,Fe)PO_4_ demonstrates high HER activity and durability in alkaline natural seawater (1 M KOH + seawater), delivering a current density of 10 mA/cm^2^ at an overpotential of 137 mV. Furthermore, the measured potential of the electrocatalyst ((Co,Fe)PO_4_) at a constant current density of −100 mA/cm^2^ remains very stable without noticeable degradation for 72 h during the continuous operation in alkaline natural seawater, demonstrating its suitability for seawater applications. Furthermore, an alkaline seawater electrolyzer employing the non-precious-metal catalysts demonstrates better performance (1.625 V at 10 mA/cm^2^) than one employing precious metal ones (1.653 V at 10 mA/cm^2^). The non-precious-metal-based alkaline seawater electrolyzer exhibits a high solar-to-hydrogen (STH) efficiency (12.8%) in a commercial silicon solar cell.

## 1. Introduction

Hydrogen is a next-generation energy source that can solve environmental pollution and the energy-depletion crisis [1,2]. Among the various methods for producing hydrogen, electrochemical water splitting represents an ecofriendly, sustainable, and efficient route. To electrochemically produce hydrogen, enormous efforts have been devoted to the development of highly active electrocatalysts for water splitting in acidic or alkaline electrolytes containing high-purity fresh water. However, with the increasing demand for high-purity fresh water owing to the development of water splitting through electrolysis, the possibility of challenges, such as water distribution, must be considered [3,4]. Thus, the electrolysis of seawater is a promising alternative for mitigating the challenges accompanying the supply of high-purity freshwater. Seawater is the most abundant source of water resources on Earth; it can be employed as an inexpensive electrolyte for electrochemical water splitting [5]. However, despite these advantages, the side reactions caused by the chlorine ions (Cl^−^) in seawater prevent seawater electrolysis [6,7,8,9]. Recently, it has been reported that the selectivity of the oxygen evolution reaction (OER) can be improved by changing the thermodynamic potential of the chlorine evolution reaction (ClER) via the adjustment of the pH of the seawater; thus, many ongoing studies have focused on developing catalysts for OER [10,11,12]. However, since the hydrogen evolution reaction (HER) is a critical reaction for generating hydrogen energy, it is necessary to develop catalysts for HER toward alkaline seawater splitting [13,14].

Generally, Pt-based precious metal catalysts are considered the best for HER. However, their practical/industrial applications are hampered by their scarcity and expensiveness [15,16,17]. Therefore, numerous studies have been conducted to overcome this and explore non-precious-metal alternatives. So far, many transition-metal-based catalysts, i.e., oxides [18,19,20], hydroxides [21,22,23], sulfides [24,25,26], nitride [27,28,29], selenides [30,31], boride [32,33], chalcogenide [34,35], and phosphides/phosphate [36,37,38,39], have been developed. Among them, transition metal phosphate/phosphide (TMP) showed most effective catalytic activity for HER [40,41,42,43,44].

In this study, we developed cobalt–iron–phosphate (Co,Fe)PO_4_ as HER electrocatalysts for alkaline seawater splitting. (Co,Fe)PO_4_ was synthesized on the surface of (Co,Fe)_3_O_4_ via a phosphidation-based chemical transformation reaction. The change in the local charge-density distribution through phosphidation lowered the energy barrier of HER, thus improving the HER activity. Further, an alkaline seawater electrolyzer employing the non-precious-metal catalysts demonstrated better performance than one employing a precious-metal catalyst. The high performance of the non-precious-metal-based seawater electrolyzer ensured its operation in seawater electrolysis with high efficiency employing commercial silicon solar cells.

## 2. Materials and Methods

### 2.1. Synthesis of (Co,Fe)OOH on Iron Foam

A (Co,Fe)OOH sample was synthesized via galvanic corrosion and grown directly on an iron foam. Before the synthesis, a piece of the iron foam (2 cm × 3 cm, Alantum Co., Seongnam-City, Korea) was first etched with 1 M HCl for 15 min to remove the surface oxide layer, after which it was washed with acetone, ethanol, and deionized water under ultrasonication for 10 min. Thereafter, the washed iron foam was immersed in 70 mL of an aqueous solution containing 3.0 mM cobalt chloride hexahydrate (CoCl_2_·6H_2_O, Sigma-Aldrich Inc., St. Louis, MO, USA) for 4 h with stirring at room temperature (25 °C). After the galvanic corrosion reaction, the (Co,Fe)OOH on the iron foam sample was thoroughly rinsed with ethanol and deionized water, followed by drying overnight in a convection oven at 70 °C. This sample was named (Co,Fe)OOH.

### 2.2. Synthesis of (Co,Fe)_3_O_4_ and (Co,Fe)PO_4_

The prepared (Co,Fe)OOH was converted into a (Co,Fe)_3_O_4_ sample via calcination for 2 h in the air at 500 °C and a heating rate of 5 °C/min employing a tube furnace. The (Co,Fe)_3_O_4_ sample, which was named (Co,Fe)_3_O_4_, was obtained after cooling to room temperature.

The (Co,Fe)PO_4_ sample was synthesized via a phosphidation process. Briefly, (Co,Fe)_3_O_4_ and 2.0 g of sodium hypophosphite (NaH_2_PO_4_, Sigma-Aldrich Inc., St. Louis, MO, USA) were placed in two separate ceramic boats in a tube furnace. Next, NaH_2_PO_4_ and (Co,Fe)_3_O_4_ were placed at the upstream and downstream sides of the Ar gas flow, respectively. Subsequently, the tube furnace was heated for 2 h to 500 °C in Ar atmosphere at a heating rate of 5 °C/min and air cooled to room temperature. The (Co,Fe)PO_4_ sample, which was obtained via the phosphidation of (Co,Fe)_3_O_4_ for 2 h, was named (Co,Fe)PO_4._

### 2.3. Characterization of Physical Properties

X-ray diffraction (XRD) patterns were recorded on an X-ray diffractometer (Ultima IV, Rigaku, Tokyo, Japan) employing a Cu-Kα radiation source over the 2θ range of 10°–90° at a scan rate of 2°/min. The surface morphologies of the samples were examined by field-emission scanning electron microscopy (FE-SEM, MIRA 3, TESCAN, Brno, Czechia). FE-transmission electron microscopy (FE-TEM), high-resolution TEM (HR-TEM), selected area electron diffraction (SAED), and elemental distribution spectroscopy (EDS) were performed on a TALOS F200X (Thermo Fisher Scientific, Waltham, USA). Further, the chemical states were investigated by X-ray photoelectron spectroscopy (XPS, K-Alpha^+^ XPS System, Thermo Fisher Scientific, Waltham, USA).

### 2.4. Electrochemical Characterization

The electrochemical properties of the electrocatalysts were investigated using a potentiostat (VersaSTAT 4, AMETEK, Oak Ridge, USA) in a three-electrode cell at room temperature. The synthesized (Co,Fe)OOH, (Co,Fe)_3_O_4_, and (Co,Fe)PO_4_ electrocatalysts were employed as the working electrode with dimensions of 1 cm × 1 cm. Hg/HgO (1 M KOH) and a graphite rod were employed as the reference and counter electrodes for the HER, respectively. The polarization curves for the HER activity were recorded via linear sweep voltammetry (LSV) at a scan rate of 1 mV/s in N_2_-purged 1 M KOH, 1 M KOH + 0.5 M NaCl, and 1 M KOH + seawater as the electrolyte. Real seawater was collected from the sea of Haeundae (Busan, Korea). The recorded potentials were converted into reversible hydrogen electrode (RHE) according to Nernst’s equation (*E_RHE_* = *E_Hg/HgO_* + 0.0591 × pH + 0.098). All the electrochemical tests were performed with 90% iR compensation, and the Tafel slopes were measured from the corresponding polarization curves. Electrochemical impedance spectroscopy (EIS) was performed at an overpotential of −0.25 V_RHE_ for HER in the frequency range from 100 kHz to 0.01 Hz with an amplitude of 10 mV. The double-layer capacitance (C*_dl_*) was estimated in the 1 M KOH solution via cyclic voltammetry (CV) at different scan rates (10, 20, 40, 80, and 160 mV/s) in the non-faradaic region. The durability tests for HER were performed at a constant current density of −100 mA/cm^2^ for 72 h. The Faradaic efficiency (FE) was determined via the water displacement method. The volume of the generated H_2_ was measured by collecting the amount of H_2_ gas at a constant current density of 50 mA/cm^2^. To prepare the Pt/C noble metal electrocatalysts for comparison, an ink solution was fabricated by mixing commercial Pt/C powder (20 mg), 5 wt.% Nafion solution (100 µL), and ethanol (900 µL). Thereafter, the ink solution was coated onto the surface of an iron foam (1 cm × 1 cm) after ultrasonic dispersion for 15 min. The loading mass of Pt/C was ~3 mg/cm^2^.

## 3. Results and Discussion

**Figure 1:**
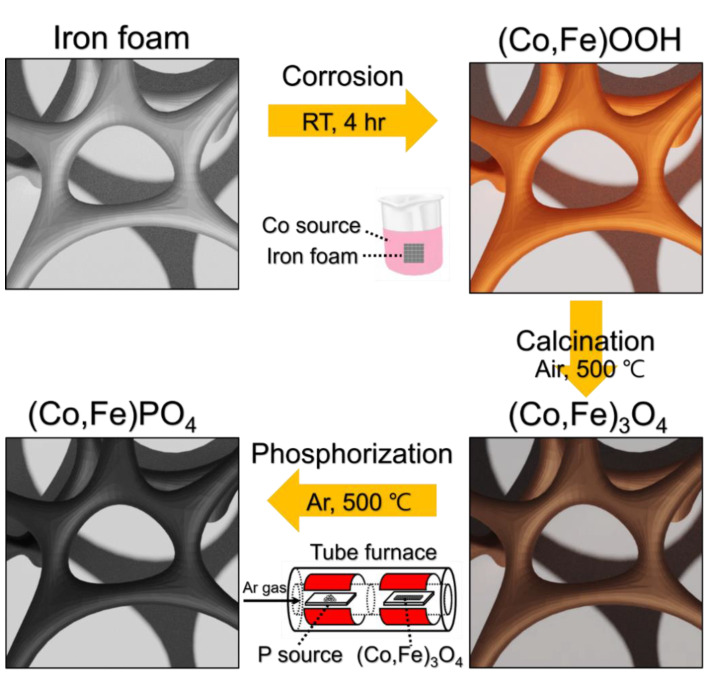
Schematic for synthesizing (Co,Fe)PO_4_ on the iron foam.

Figure 1 shows the schematic for synthesizing the (Co,Fe)PO_4_ electrocatalysts. Firstly, (Co,Fe)OOH was directly synthesized on the iron foam via surface corrosion in a CoCl_2_ aqueous solution at room temperature. The prepared (Co,Fe)OOH was converted into (Co,Fe)_3_O_4_ through calcination, after which the nanoneedle shape of (Co,Fe)PO_4_ was synthesized through phosphidation.

To determine the crystalline structures of the synthesized (Co,Fe)OOH, (Co,Fe)_3_O_4_, and (Co,Fe)PO_4_ electrocatalysts, the XRD patterns were obtained (Figure S1). The diffraction peaks of the (Co,Fe)OOH sample appeared at 2θ = 27.0°, 36.4°, 46.9°, 60.8°, and 79.6°, and were indexed to the (021), (130), (150), (132), and (202) planes, respectively, of iron oxyhydroxide (FeOOH, JCPDS # 01-073-2326). The diffraction peaks of the (Co,Fe)_3_O_4_ sample that appeared at 2θ = 18.3°, 30.1°, 35.5°, 37.1°, 43.1°, 57.0°, and 62.6° were indexed to the (111), (220), (311), (222), (400), (511), and (440) planes, respectively, of iron oxide (Fe_3_O_4_, JCPDS # 01-075-0033). However, for the (Co,Fe)PO_4_ sample, both the iron phosphate and iron oxide phases were discerned, corresponding to FePO_4_ (JCPDS # 00-029-0715) and Fe_3_O_4_ (JCPDS # 01-075-0033). This result indicated the hybrid structure of (Co,Fe)_3_O_4_ and (Co,Fe)PO_4_. The peaks of Fe_3_O_4_ exhibited almost identical diffraction peaks with the (Co,Fe)_3_O_4_ sample, and the peaks from the (100) and (102) planes of FePO_4_ were observed at 20.3° and 25.8°, respectively. Generally, chemical transformation reactions, such as sulfurization, phosphidation, and selenization, also occurred at the surface [45,46,47]. Therefore, after phosphidation, the outer region of (Co,Fe)_3_O_4_ was converted into (Co,Fe)PO_4_, and the inner region of (Co,Fe)_3_O_4_ did not participate in the chemical transformation reaction [48,49].

**Figure 2:**
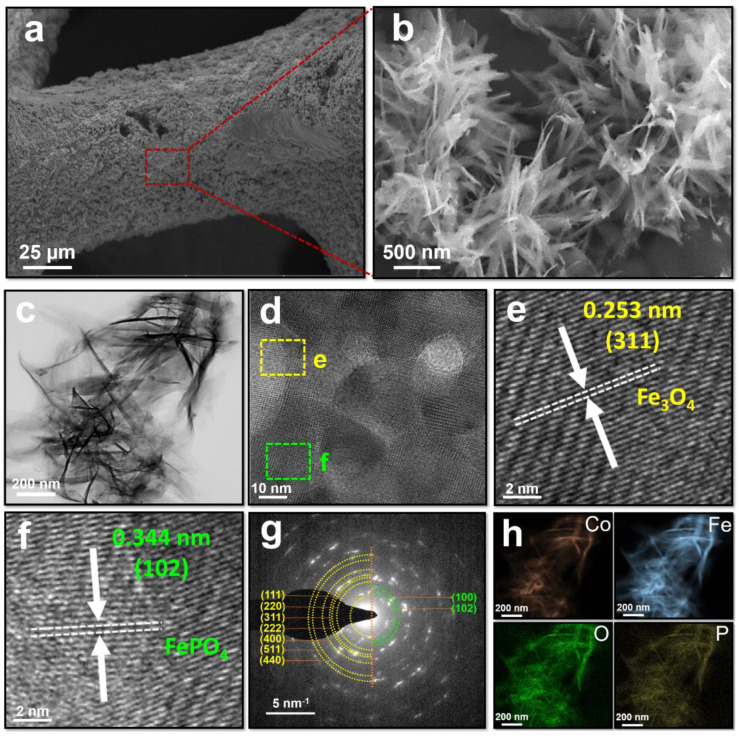
Characterization of (Co,Fe)PO_4_. (**a**) Low- and (**b**) high-magnification SEM images, (**c**) TEM image, (**d**–**f**) HR-TEM image, (**g**) SAED ring patterns, and (**h**) TEM-EDS mapping images of (Co,Fe)PO_4_. Color codes: Co (red), Fe (blue), O (green), and P (yellow).

The surface morphologies of (Co,Fe)OOH, (Co,Fe)_3_O_4_, and (Co,Fe)PO_4_ were observed via the FE-SEM images. (Co,Fe)OOH exhibited thin nanosheets (Figure S2), and (Co,Fe)_3_O_4_, which was obtained by calcining (Co,Fe)OOH, exhibited a nanoneedle morphology (Figure S3). Interestingly, (Co,Fe)PO_4_ and (Co,Fe)_3_O_4_ exhibited almost the same surface morphologies even after phosphidation (Figure 2a,b). Particularly, the shape of the nanoneedles could extensively increase the concentrations of the reactants in the active sites and enhance local electric fields that promote the intrinsic catalytic activity [50]. Therefore, the shape of (Co,Fe)PO_4_ is suitable for electrochemical water splitting.

The TEM images were obtained to confirm the surface morphology and phase information. (Co,Fe)OOH exhibited a nanosheet morphology (Figure S4a). After calcination, (Co,Fe)_3_O_4_ exhibiting a nanoneedle shape was obtained (Figure S5a) owing to the escape of the water molecules in (Co,Fe)OOH during calcination. Interestingly, the nanoneedle shape was maintained well after phosphidation (Figure 2c). Furthermore, the phase information was obtained from the SAED patterns. The ring patterns of (Co,Fe)OOH were indexed to the planes of the (021), (130), (150), and (202) reflections of FeOOH (inset of Figure S4a). Additionally, the ring patterns of (Co,Fe)_3_O_4_ were indexed to the planes of the (111), (220), (311), and (222) reflections of Fe_3_O_4_ (inset of Figure S5a). Further, the elemental distributions of (Co,Fe)OOH and (Co,Fe)_3_O_4_ were uniform (Figures S4b and S5b). The lattice fringes and ring pattern of (Co,Fe)PO_4_ exhibited both Fe_3_O_4_ and FePO_4_ patterns, which are consistent with the XRD results (Figure 2d–g). The EDS mapping of (Co,Fe)PO_4_ confirmed that each element was uniformly distributed therein (Figure 2h). The EDX spectrum in the collected area is shown in Figure S7. Interestingly, the high-magnification TEM-EDS mapping images revealed that elemental P was mainly distributed in the outer region and that elemental Co and Fe were mainly distributed in the inner region. Additionally, elemental O was uniformly distributed in the inner and outer regions (Figure S6). These results indicated that the chemical transformation reaction proceeded on the surface.

**Figure 3:**
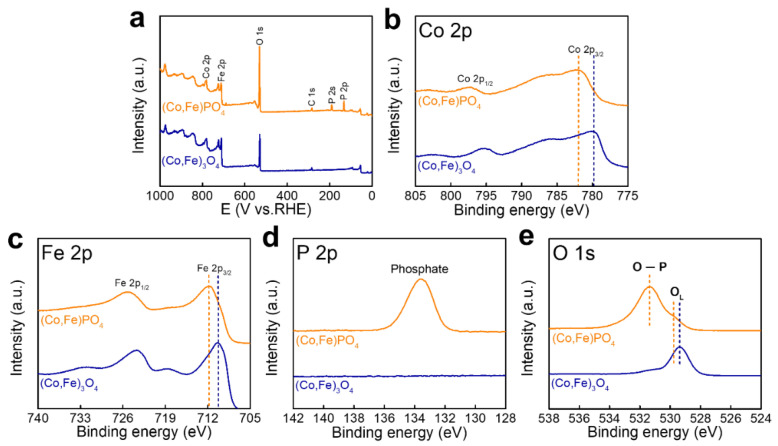
Analyses of the chemical states. (**a**) Full XPS survey spectra of (Co,Fe)_3_O_4_ and (Co,Fe)PO_4_, (**b**) Co 2p, (**c**) Fe 2p, (**d**) P 2p, and (**e**) O 1s.

XPS analysis was performed to investigate the surface chemical states of (Co,Fe)_3_O_4_ and (Co,Fe)PO_4_ (Figure 3). Figure 3a shows the full XPS survey spectra of (Co,Fe)_3_O_4_ and (Co,Fe)PO_4_, which clearly confirmed the existence of Co, Fe, P and O. Figure 3b–e shows the HR-XPS profiles of Co, Fe, P and O. Notably, the binding energies of Co 2p and Fe 2p shifted along a higher direction after phosphidation. Additionally, the binding energy of O 1s shifted along a higher direction. These observations indicated that electrons were transferred from (Co,Fe)_3_O_4_ to (Co,Fe)PO_4_ in the hybrid (Co,Fe)_3_O_4_ and (Co,Fe)PO_4_ structures [51]. This changed local charge-density distribution was expected to reduce the energy barrier of HER, thus facilitating the adsorption and desorption processes between the reactant and resultant molecules [52,53,54,55].

**Figure 4:**
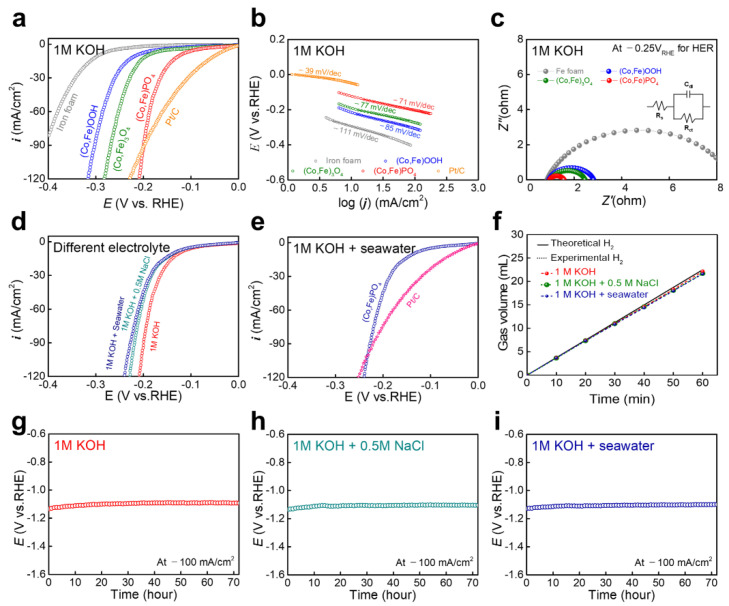
Electrochemical analyses for HER. (**a**) Forward scan polarization curves for HER in 1 M KOH. (**b**) Tafel plots for HER and (**c**) P-EIS at −0.25 V_RHE_ for HER. Polarization curves for HER in (**d**) different electrolytes and (**e**) 1 M KOH + seawater. (**f**) The FEs of (Co,Fe)PO_4_ in 1 M KOH and 1 M KOH + seawater at 50 mA/cm^2^. Durability test at a constant current density of −100 mA/cm^2^ for 72 h in (**g**) 1.0 M KOH, (**h**) 1.0 M KOH + 0.5 M NaCl, and (**i**) 1.0 M KOH + seawater.

LSV was performed to measure the HER activity in a 1 M KOH solution (Figure 4a). For comparison, Pt/C, which is a benchmark precious metal electrocatalyst for HER, was tested; it exhibited a low overpotential of 48 mV at −10 mA/cm^2^. Moreover, (Co,Fe)OOH and (Co,Fe)_3_O_4_ exhibited overpotentials of 215 and 191 mV at −10 mA/cm^2^, respectively. Interestingly, (Co,Fe)PO_4_, obtained through phosphidation, exhibited a significantly reduced overpotential (122 mV at −10 mA/cm^2^). Although the overpotential of (Co,Fe)PO_4_ was relatively higher compared with that of Pt/C, it still outperformed Pt/C at a high current density. This result is because the nanoneedle shape increased the concentration of the reactant in the active site and concurrently enhanced the local electric field [50]. The Tafel plots were calculated to elucidate the electrocatalytic kinetics. Figure 4b shows the Tafel slopes that were derived from the HER polarization curves. (Co,Fe)PO_4_ displayed a lower Tafel slope (−71 mV/dec) compared with those of (Co,Fe)_3_O_4_ (−77 mV/dec), (Co,Fe)OOH (−85 mV/dec), and the bare iron foam (−111 mV/dec). These results indicate that (Co,Fe)PO_4_ exhibited faster reaction kinetics for HER. Generally, HER proceeds via two different reaction routes: the Volmer–Heyrovsky and Volmer–Tafel mechanisms [56,57]. Considering the Tafel slope of (Co,Fe)PO_4_, it was inferred that (Co,Fe)PO_4_ followed the Volmer–Heyrovsky mechanism [58]. The electrochemically active surface area (ECSA) was estimated employing C*_dl_* that was derived via CV in the non-Faradaic region (Figures S8 and S9). (Co,Fe)PO_4_ and (Co,Fe)OOH exhibited the highest and the smallest C_dl_ values, respectively, indicating that (Co,Fe)PO_4_ exhibited the largest ECSA. Since ECSA was directly proportional to the number of active sites, as well as the efficiency of the mass and charge transports of catalysts, the largest ECSA of (Co,Fe)PO_4_ indicated that it exhibited the most active sites, as well as the most effective mass- and charge-transport capabilities, which imparted it with the best HER activity [59]. EIS was performed to confirm the charge-transfer resistances of (Co,Fe)OOH, (Co,Fe)_3_O_4_, and (Co,Fe)PO_4_. Figure 4c shows the Nyquist plots, which were fitted into an inserted equivalent-circuit model, where *R_s_* is the solution resistance and *R_ct_* is the charge-transfer resistance [60]. (Co,Fe)PO_4_ exhibited the smallest semicircular diameter (*R_ct_* = 0.60 Ω), indicating the lowest *R_ct_* compared with those of (Co,Fe)_3_O_4_ (*R_ct_* = 1.44 Ω) and (Co,Fe)OOH (*R_ct_* = 1.89 Ω). To confirm the catalytic activity for HER in alkaline seawater, the LSV graphs were measured for different electrolytes (Figure 4d): alkaline solution (1 M KOH), artificial alkaline seawater (1 M KOH + 0.5 M NaCl), and real alkaline seawater (1 M KOH + seawater). The overpotentials of (Co,Fe)PO_4_ were 134 and 137 mV at a current density of 10 mA/cm^2^ in the 1 M KOH + 0.5 M NaCl and 1 M KOH + seawater electrolytes, respectively. The HER activities of (Co,Fe)PO_4_ in 1 M KOH + 0.5 M NaCl and 1 M KOH + seawater were slightly lower than that of 1 M KOH. In the seawater environment, including real alkaline seawater, the HER activity was reduced owing to the blocking of Mg(OH)_2_ or Ca(OH)_2_ by the active site via precipitation [61]. Furthermore, impurities, such as bacteria, in the seawater interfered with the electrochemical reaction [6]. Compared with Pt/C, (Co,Fe)PO_4_ exhibited better HER activity in real alkaline seawater, as well as the 1 M KOH solution, at a high current density (Figure 4e). FE was measured by collecting the generated H_2_ gas via the water displacement method at a constant current density of −50 mA/cm^2^ (Figure 4f). The FEs of (Co,Fe)PO_4_ in 1 M KOH, 1 M KOH + NaCl, and 1 M KOH + seawater were still >98.6%, 96.5%, and 96.3% after 60 min, indicating that most of the electrons that participated in the reaction were consumed during HER. In addition to the catalytic activity, durability is also an essential factor for evaluating the performance of catalysts in practical applications [62,63]. The long-term stability of (Co,Fe)PO_4_ for HER was tested by measuring the potentials in different electrolytes for over 72 h at a constant current density of −100 mA/cm^2^ (Figure 4g–i). The measured potential indicated high stability during the continuous operation in all electrolytes (no noticeable deterioration was observed), demonstrating its excellent HER durability.

**Figure 5:**
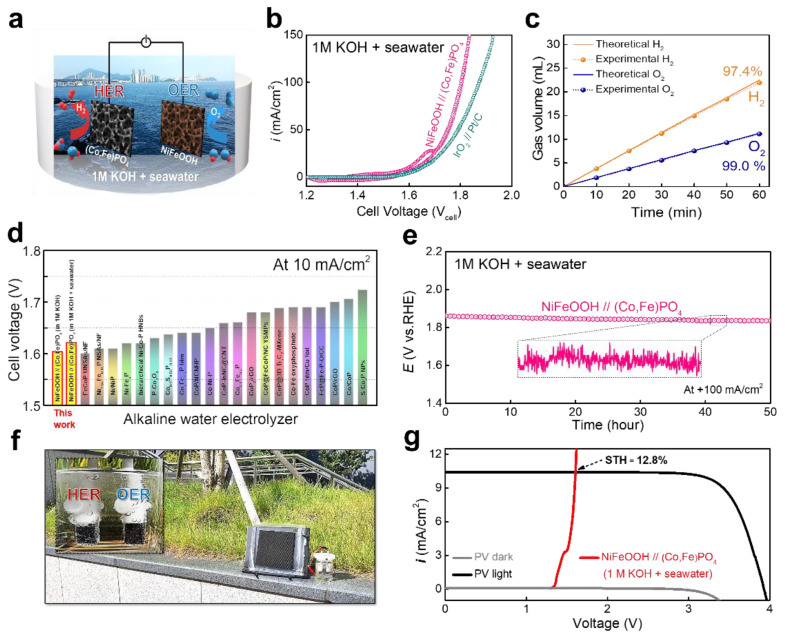
Overall seawater splitting. (**a**) Schematic of the alkaline seawater electrolyzer. (**b**) Polarization curves of the NiFeOOH//(Co,Fe)PO_4_ electrolyzer compared with that of the IrO_2_//Pt/C noble-metal electrolyzer in the 1 M KOH + seawater electrolyte. (**c**) FE at +50 mA/cm^2^ for the overall seawater splitting. (**d**) Comparison of the performances of the alkaline water electrolyzer. (**e**) Durability test at a constant current density of +100 mA/cm^2^ for 50 h in 1 M KOH + seawater. (**f**) Photograph of the setup of the solar-driven overall seawater-splitting system. (**g**) Current density–voltage (J–V) curves under dark and simulated AM 1.5G 100 mW/cm^2^ illumination for commercial silicon solar cell combined with the seawater electrolyzer.

Regarding the full-cell applications, a two-electrode alkaline water electrolyzer, which was assembled with (Co,Fe)PO_4_ and NiFeOOH as the cathode and anode, respectively, was set up for overall seawater splitting employing alkaline natural seawater (1 M KOH + seawater) (Figure 5a). NiFeOOH, which is known as the best OER catalyst, was prepared, following a reported method, [64] and the polarization curve of the OER activity is shown in Figure S10. To avoid interference with the oxidation current, a cell voltage of 10 mA/cm^2^ was measured via reverse-swept CV [65]. Interestingly, Figure 5b shows that the NiFeOOH//(Co,Fe)PO_4_ electrolyzer exhibited excellent activity in this two-electrode system for overall seawater splitting in 1 M KOH + seawater. This electrolyzer required low voltages of 1.625 (η = 395 mV at 10 mA/cm^2^), 1.749 (η = 519 mV at 50 mA/cm^2^), and 1.801 V (η = 571 mV at 100 mA/cm^2^) in 1M KOH + seawater, demonstrating better overall water-splitting performance compared with the IrO_2_//Pt/C precious metal electrolyzer in both 1 M KOH (Figure S11) and 1 M KOH + seawater (Figure 5b). The performance of the NiFeOOH//(Co,Fe)PO_4_ electrolyzer in 1 M KOH + seawater was comparable with or outperformed the recently reported electrolyzer that was based on the transition metal electrolyzer (Figure 5d). The FE of the NiFeOOH//(Co,Fe)PO_4_ alkaline water electrolyzer was calculated by collecting the generated O_2_ and H_2_ gases for 60 min from each electrode at a constant current density of 50 mA/cm^2^ in 1 M KOH + seawater (Figure 5c). The measured FE demonstrated high energy conversion rates of 97.4% and 99.0% for HER and OER in 1M KOH + seawater, respectively. Moreover, the NiFeOOH//(Co,Fe)PO_4_ electrolyzer in 1 M KOH + seawater exhibited excellent durability. To confirm the long-term stability of the (Co,Fe)PO_4_ electrolyzer, the measured voltage at a constant current density of +100 mA/cm^2^ remained very stable without any noticeable deterioration for 50 h in the 1 M KOH + seawater electrolytes (Figure 5e). The durability test, which was conducted in the 1 M KOH electrolyte at a constant current density (+100 mA/cm^2^) for 50 h, further confirmed the high stability (Figure S12). These results demonstrate that the NiFeOOH//(Co,Fe)PO_4_ alkaline water electrolyzer exhibited a high potential for application as a high-efficiency and durable seawater electrolyzer in natural seawater environments. In order to confirm the change in the morphology and phase, the SEM image and XRD patterns were presented in Figure S13. The surface morphology after durability test was well maintained. In addition, the XRD pattern showed an almost identical pattern to that of (Co,Fe)PO_4_ before the durability test. These results indicate that the morphology and crystal structure of (Co,Fe)PO_4_ were still maintained after the durability test.

Furthermore, driving the electrolysis with natural solar power without artificial current is an ecofriendly and attractive method for conserving the cost of hydrogen production. Thus, the NiFeOOH//(Co,Fe)PO_4_ seawater electrolyzer was combined with a commercial silicon solar cell to set up a photo-assisted water-splitting system (Figure 5f), after which the overall seawater splitting performance was evaluated in the 1 M KOH + seawater electrolyte under natural sunlight. Figure 5g shows the J–V curve of a commercial silicon solar cell, and the solar-to-hydrogen (STH) efficiency was calculated from the intersection of the power curve of the solar cell and the polarization curve of the electrolyzer [8], yielding an STH of 12.8%. When this photo-assisted seawater splitting device was driven under natural sunlight, the continuous release of H_2_ and O_2_ bubbles from both electrodes was clearly observed, confirming the successful production of H_2_ (inset of Figure 5e). Therefore, the photo-assisted seawater splitting system developed in this study could be applied to cost-effective hydrogen production in the seawater-splitting industry.

## 4. Conclusions

In summary, a non-precious-metal catalyst, (Co,Fe)PO_4_, was developed as an HER electrocatalyst for alkaline seawater electrolysis. (Co,Fe)PO_4_ demonstrated impressive HER activity with a low overpotential of 134 mV at −10 mA/cm^2^ in 1 M KOH + seawater, as well as excellent durability. The nanoneedle shape of (Co,Fe)PO_4_ enhanced the local electric field, and its electronic structure, which was modified via phosphidation, enhanced the HER activity. The assembled seawater electrolyzer employing the non-precious-metal catalysts delivered excellent performance (1.625 V in 1 M KOH + seawater), which surpassed those of precious-metal-based electrolyzers. Further, the combination of the non-precious-metal-based electrolyzer with a commercial silicon solar cell successfully generated H_2_ gas under natural sunlight in alkaline natural seawater. This study demonstrates that non-precious-metal-based electrolyzers can outperform precious-metal-based ones, indicating that cost-effective hydrogen production without artificial current is feasible with commercial silicon solar cells.

## Data Availability

Data are contained within the article.

## Supplementary Materials

The following are available online at https://www.mdpi.com/article/10.3390/nano11112989/s1, Figure S1: XRD patterns of (Co,Fe)PO_4_, (Co,Fe)_3_O_4_, and (Co,Fe)OOH, Figure S2: SEM images of (Co,Fe)OOH on iron foam, Figure S3: SEM images of (Co,Fe)_3_O_4_ on iron foam, Figure S4: (a) TEM image of (Co,Fe)OOH with selected area electron diffraction (SAED) ring patterns (insert), and (b) TEM-EDS mapping images of (Co,Fe)OOH, Figure S5: (a) TEM image of (Co,Fe)_3_O_4_ with selected area electron diffraction (SAED) ring patterns (insert), and (b) TEM-EDS mapping images of (Co,Fe)_3_O_4_, Figure S6: TEM-EDS mapping of (Co,Fe)PO_4_, Figure S7: EDX spectrum of (Co,Fe)PO_4_, Figure S8: Cyclic voltammetry curves of (a) (Co,Fe)OOH, (b) (Co,Fe)_3_O_4_, and (c) (Co,Fe)PO_4_ in non-Faradaic region at different scan rates 10–160 mV/s, Figure S9: Double layer capacitance (C*_dl_*) of (a) (Co,Fe)OOH, (b) (Co,Fe)_3_O_4_, and (c) (Co,Fe)PO_4_, Figure S10: Polarization curves of NiFeOOH electrocatalysts for OER in 1 M KOH. To avoid interference with the oxidation current, a cell voltage of 10 mA/cm^2^ was measured at the reverse-swept cyclic voltammetry, Figure S11: Polarization curves of NiFeOOH//(Co,Fe)PO_4_ electrolyzer for overall seawater splitting compared to IrO_2_//Pt/C noble metal electrolyzer in 1 M KOH electrolyte. To avoid interference with the oxidation current, a cell voltage of 10 mA/cm^2^ was measured at the reverse-swept cyclic voltammetry, Figure S12: Durability test of NiFeOOH//(Co,Fe)PO_4_ electrolyzer conducted at constant current density of +100 mA/cm^2^ for 50 h in 1 M KOH electrolyte, Figure S13: (a) SEM image and (b) XRD results of (Co,Fe)PO4 after durability test, Table S1: Comparison of overall water splitting performance of the NiFeOOH//(Co,Fe)PO_4_ with recently reported transition metal-based alkaline water electrolyzers in 1 M KOH.
