# Supplementary material for: Cobalt–Iron–Phosphate Hydrogen Evolution Reaction Electrocatalyst for Solar-Driven Alkaline Seawater Electrolyzer
Source: Nanomaterials (Basel). 2021 Nov 6;11(11):2989. doi: 10.3390/nano11112989 (PMC8624952; doi:10.3390/nano11112989)
Supplement: Supplementary file 1 [file nanomaterials-11-02989-s001.zip › nanomaterials-1439393-SI modify.pdf]

nanomaterials

Supporting information

Cobalt-Iron-Phosphate hydrogen evolution reaction electrocatalyst for solar-driven alkaline seawater electrolyzer

Chiho Kim [1], Seunghun Lee [1], Seong Hyun Kim [1], Jaehan Park [1], Shinho Kim [2], Se-Hun Kwon [1], Jong-Seong Bae [3], Yoo Sei Park [1,4,*] and Yangdo Kim [1,*]

1 Department of Materials Science and Engineering, Pusan National University, Busan 46241, Korea; chihokim@pusan.ac.kr (C.K.); basicroof@hanmail.net (S.L.); kshe1995@naver.com (S.H.K.); parkoo68@pusan.ac.kr (J.P.); sehun@pusan.ac.kr (S.-H.K.)
2 BK21 four, Innovative Graduate Education Program for Global High-tech Materials & Parts, Pusan National University, Busan 46241, Republic of Korea; shinho@pusan.ac.kr (S.K.)
3 Busan Center, Korea Basic Science Institute, Busan, Korea; jsbae@kbsi.re.kr (J.-S.B.)
4 Department of Chemical Engineering, Kansas State University, 1701A Platt St Manhattan, KS, 66506, USA
* Correspondence: qkrdbtp@pusan.ac.kr (Y.S.P); yangdo@pusan.ac.kr (Y.K.)

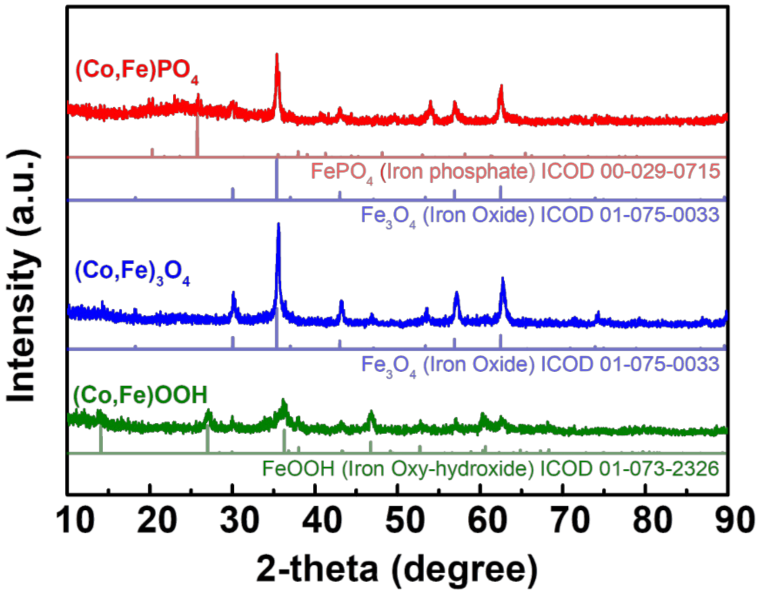

Figure S1. XRD patterns of (Co,Fe)PO_4, $(Co,Fe)_3O_4$, and (Co,Fe)OOH.

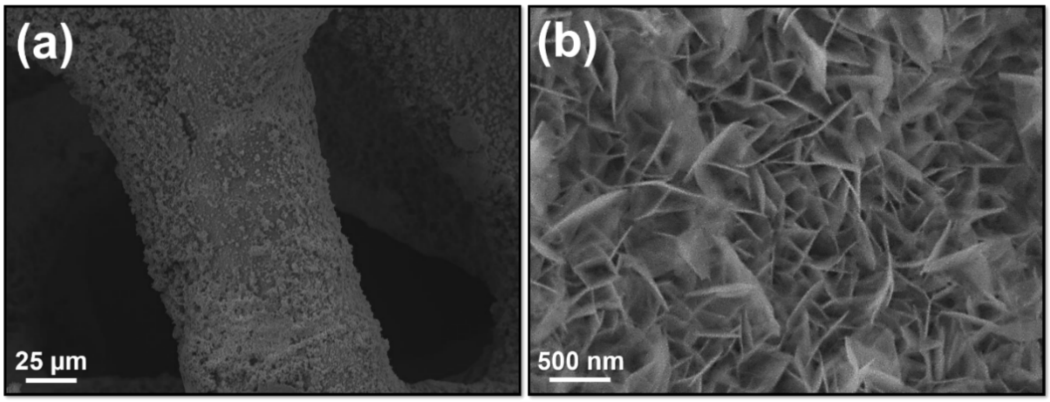

Figure S2. SEM images of (a) (Co,Fe)OOH and (b) iron foam.

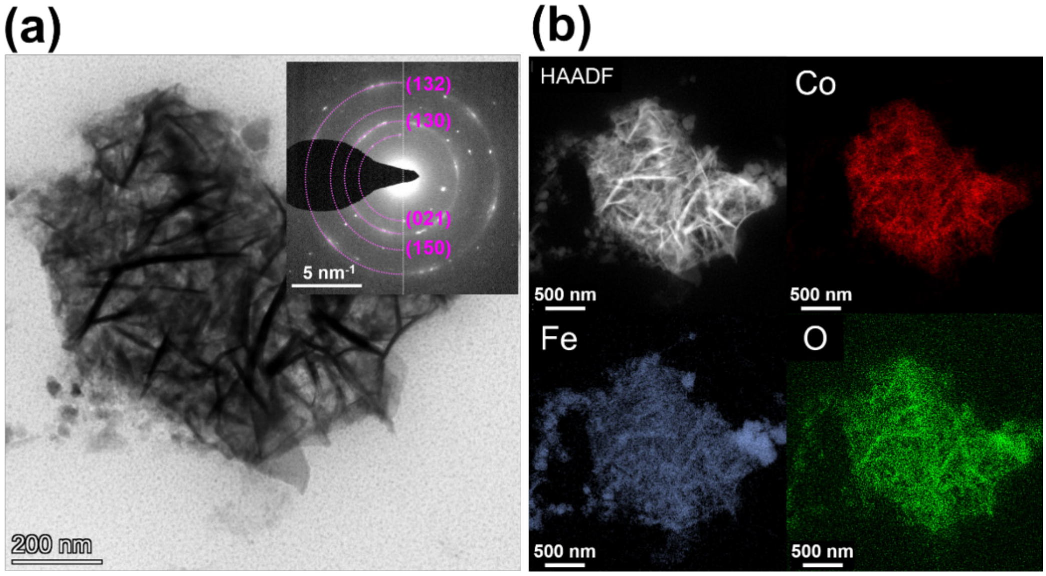

Figure S3. (a) TEM image of (Co,Fe)OOH with selected area electron diffraction (SAED) ring patterns (insert), and (b) TEM-EDS mapping images of (Co,Fe)OOH.

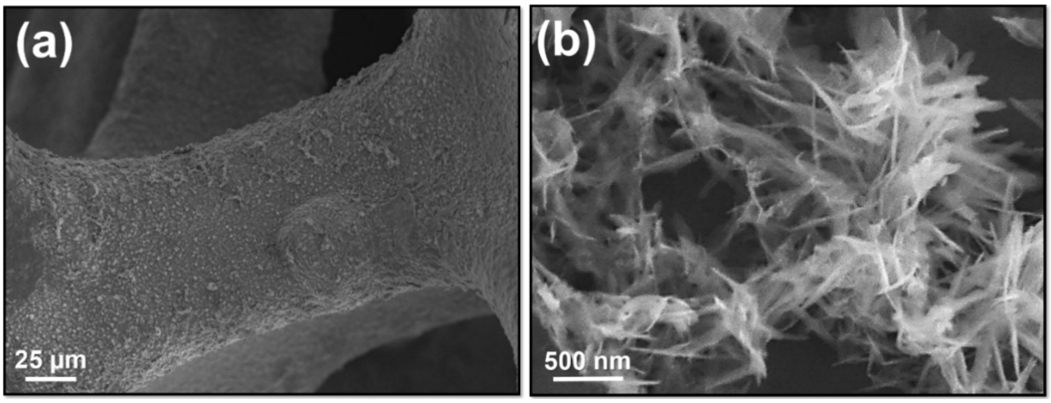

Figure S4. SEM images of (a) $(Co,Fe)_3O_4$ and (b) iron foam.

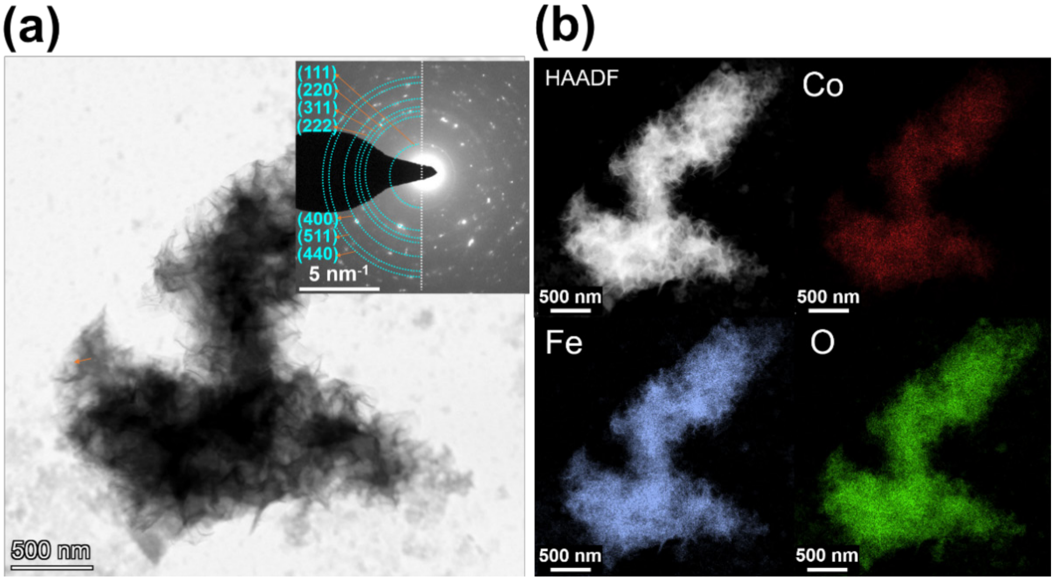

Figure S5. (a) TEM image of $(Co,Fe)_3O_4$ with selected area electron diffraction (SAED) ring patterns (insert), and (b) TEM-EDS mapping images of $(Co,Fe)_3O_4$.

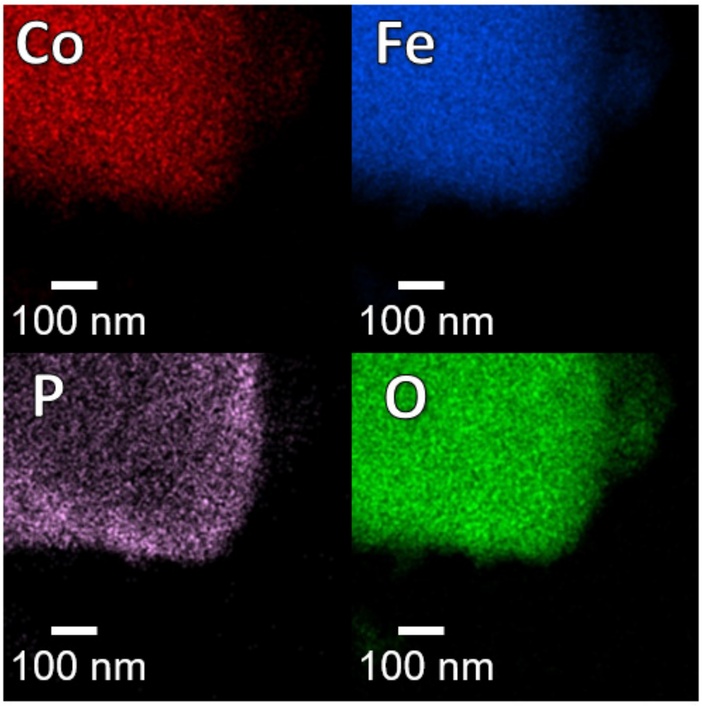

Figure S6. TEM-EDS mapping of $(Co,Fe)PO_4$.

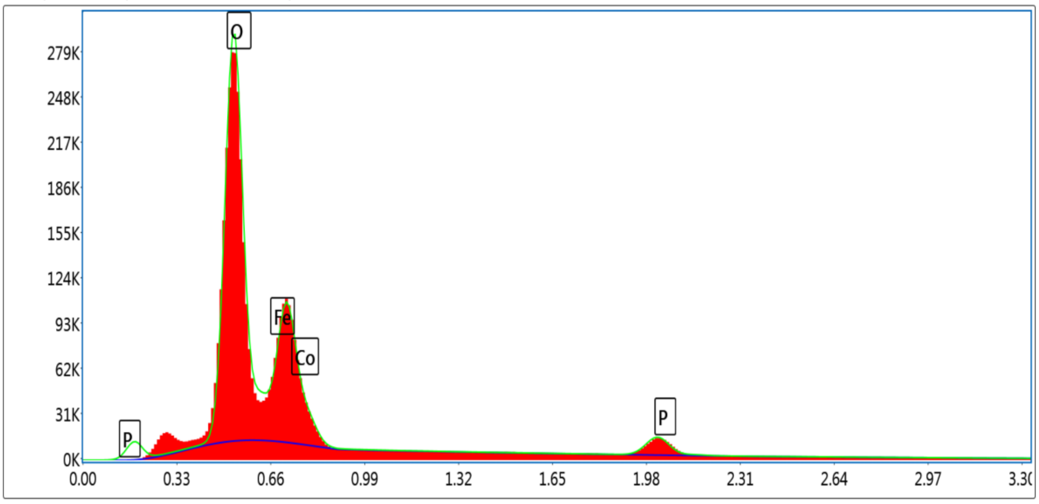

Figure S7. EDX spectrum of $(Co,Fe)PO_4$

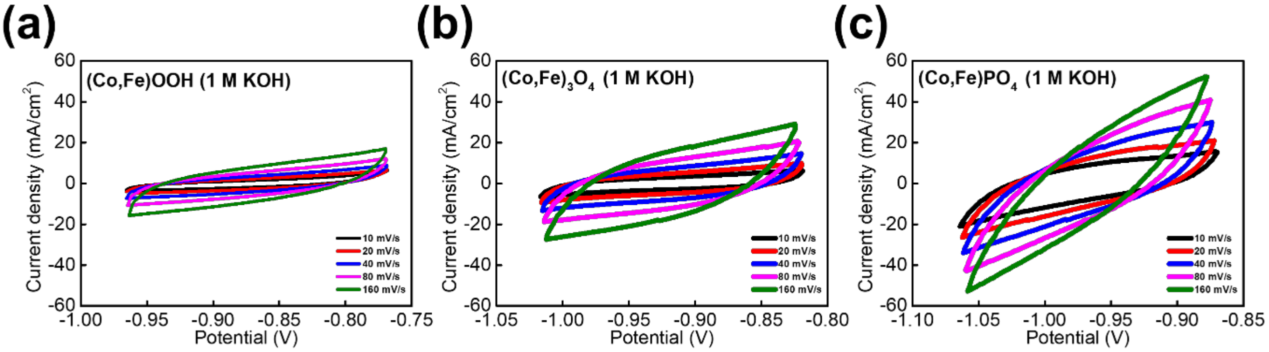

Figure S8. Cyclic voltammetry curves of (a) (Co,Fe)OOH, (b) $(Co,Fe)_3O_4$, and (c) $(Co,Fe)PO_4$ in non-Faradaic region at different scan rates 10–160 mV/s.

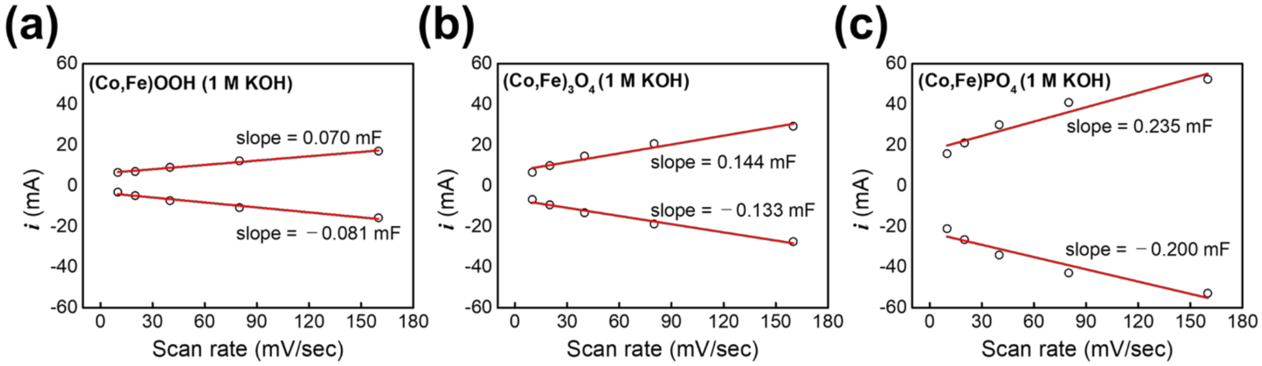

Figure S9. Double layer capacitance (C_{dl}) of (a) (Co,Fe)OOH, (b) $(Co,Fe)_3O_4$, and (c) $(Co,Fe)PO_4$.

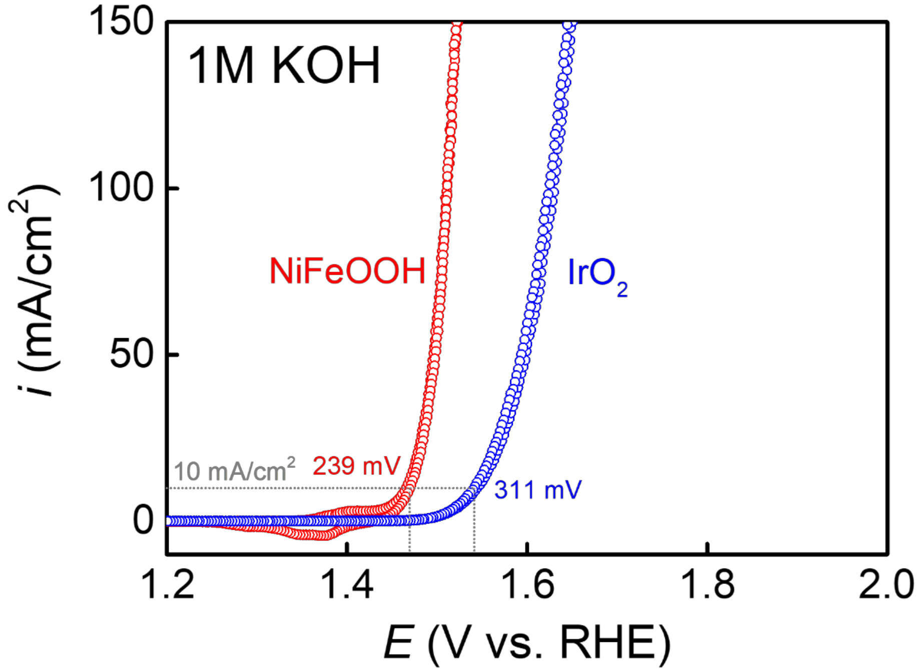

Figure S10. Polarization curves of NiFeOOH electrocatalysts for OER in 1 M KOH. To avoid interference with the oxidation current, a cell voltage of 10 mA/cm² was measured at the reverse-swept cyclic voltammetry.

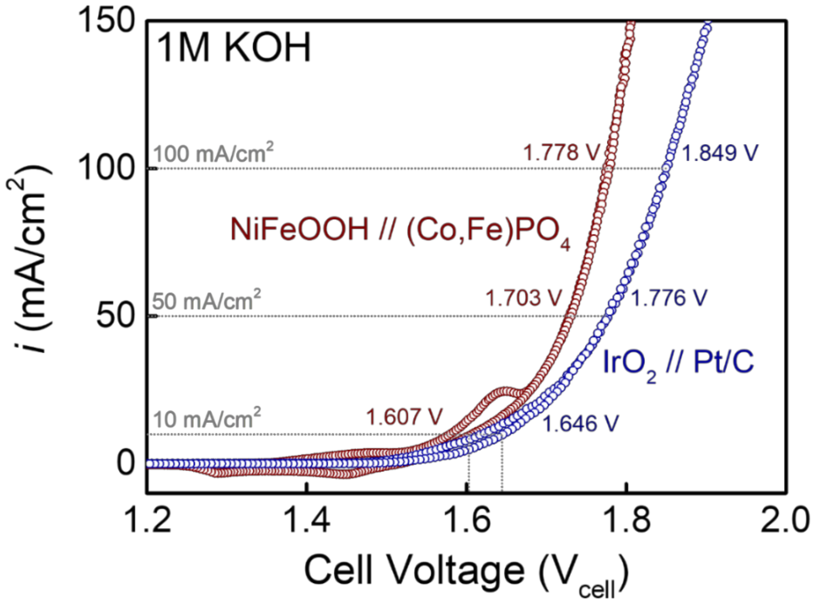

Figure S11. Polarization curves of NiFeOOH // (Co,Fe)PO_4 electrolyzer for overall seawater splitting compared to IrO_2 // Pt/C noble metal electrolyzer in 1 M KOH electrolyte. To avoid interference with the oxidation current, a cell voltage of 10 mA/cm² was measured at the reverse-swept cyclic voltammetry.

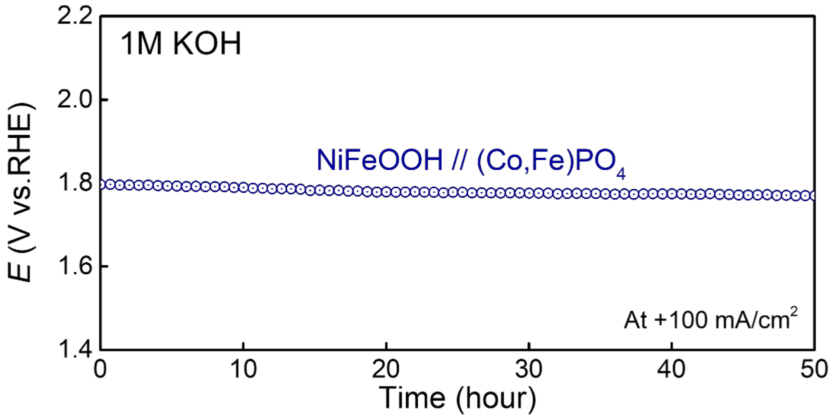

Figure S12. Durability test of NiFeOOH // (Co,Fe)PO_4 electrolyzer conducted at constant current density of +100 mA/cm^2 for 50 h in 1 M KOH electrolyte.

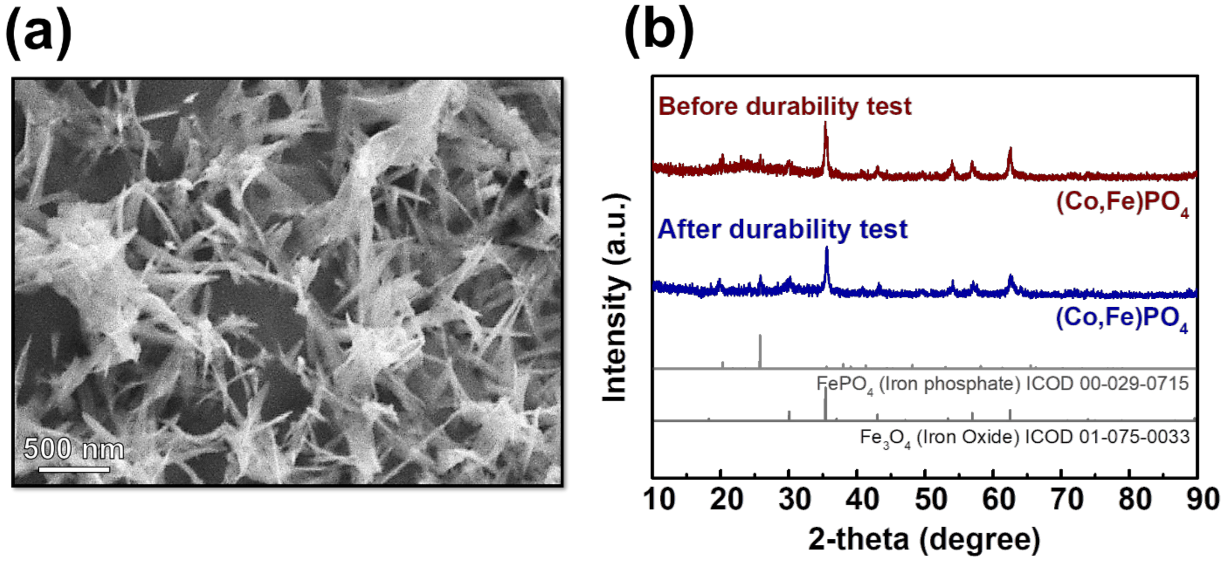

Figure S13. (a) SEM image and (b) XRD results of (Co,Fe)PO_4 after durability test.

Table S1. Comparision of overall water splitting performance of the NiFeOOH // (Co,Fe)PO_4 with recently reported transition metal-based alkaline water electrolyzers in 1 M KOH.

Catalyst	Electrolyte	η_{10} for HER (mV)	η_{10} for OER (mV)	Voltage(η_{10}) (V)	Reference
NiFeOOH // (Co,Fe)PO_4	1 M KOH	122	239	1.607	This work
NiFeOOH // (Co,Fe)PO_4	1 M KOH + seawater	136	-	1.625	This work
FeCoP UNSAs/NF	1 M KOH	188 (η_{100})	260 (η_{20})	1.6	[1]
$Ni_{1.85}Fe_{0.15}P$ NSAs/NF	1 M KOH	106	270 (η_{20})	1.61	[2]
Ni/NiP	1 M KOH	130	270 (η_{30})	1.61	[3]
Ni-Fe_xP	1 M KOH	119	267 (η_{100})	1.62	[4]
hierarchical Ni-Co-P HNBs	1 M KOH	107	270	1.62	[5]
P-Co_3O_4	1 M KOH	120	290	1.63	[6]
$Co_{0.9}S_{0.58}P_{0.42}$	1 M KOH	141	266	1.637	[7]
Co_xFe_{1-x}-P film	1 M KOH	169	290	1.64	[8]
CoP/NCNHP	1 M KOH	115	310	1.64	[9]
Co-Ni-P	1 M KOH	103	340	1.65	[10]
CoP-InNC@CNT	1 M KOH	159	270	1.659	[11]
$Co_{0.6}Fe_{0.4}P$	1 M KOH	133	298	1.661	[12]
CoP_2/rGO	1 M KOH	115	370	1.68	[13]
CoP@FeCoP/NC YSMPs	1 M KOH	141	238	1.68	[14]
CoP@3D Ti_3C_2-MXene	1 M KOH	168	290	1.688	[15]
Co-Fe oxyphosphide	1 M KOH	180	280	1.69	[16]
CoP film/Cu foil	1 M KOH	94	345	1.69	[17]
FeP@Fe-P-O/CC	1 M KOH	120	288	1.69	[18]
CoP/rGO	1 M KOH	150	340	1.70	[19]
Co/CoP	1 M KOH	193	283	1.706	[20]
S:Co_2P NPs	1 M KOH	184	310	1.724	[21]
FeCo-FeCoNi	1 M KOH	211	325	1.766	[22]

References

1. Zhou, L.; Shao, M.; Li, J.; Jiang, S.; Wei, M.; Duan, X. Two-dimensional ultrathin arrays of CoP: electronic modulation toward high performance overall water splitting. *Nano Energy* **2017**, *41*, 583-590.
2. Wang, P.; Pu, Z.; Li, Y.; Wu, L.; Tu, Z.; Jiang, M.; Kou, Z.; Amiinu, I.S.; Mu, S. Iron-doped nickel phosphide nanosheet arrays: an efficient bifunctional electrocatalyst for water splitting. *ACS applied materials & interfaces* **2017**, *9*, 26001-26007.
3. Chen, G.F.; Ma, T.Y.; Liu, Z.Q.; Li, N.; Su, Y.Z.; Davey, K.; Qiao, S.Z. Efficient and stable bifunctional electrocatalysts Ni/NixMy (M= P, S) for overall water splitting. *Advanced Functional Materials* **2016**, *26*, 3314-3323.
4. Zhang, C.; Xie, Y.; Deng, H.; Zhang, C.; Su, J.-W.; Dong, Y.; Lin, J. Ternary nickel iron phosphide supported on nickel foam as a high-efficiency electrocatalyst for overall water splitting. *International Journal of Hydrogen Energy* **2018**, *43*, 7299-7306.
5. Hu, E.; Feng, Y.; Nai, J.; Zhao, D.; Hu, Y.; Lou, X.W.D. Construction of hierarchical Ni–Co–P hollow nanobricks with oriented nanosheets for efficient overall water splitting. *Energy & Environmental Science* **2018**, *11*, 872-880.
6. Xiao, Z.; Wang, Y.; Huang, Y.-C.; Wei, Z.; Dong, C.-L.; Ma, J.; Shen, S.; Li, Y.; Wang, S. Filling the oxygen vacancies in Co 3 O 4 with phosphorus: an ultra-efficient electrocatalyst for overall water splitting. *Energy & Environmental Science* **2017**, *10*, 2563-2569.
7. Dai, Z.; Geng, H.; Wang, J.; Luo, Y.; Li, B.; Zong, Y.; Yang, J.; Guo, Y.; Zheng, Y.; Wang, X. Hexagonal-phase cobalt monophosphosulfide for highly efficient overall water splitting. *ACS nano* **2017**, *11*, 11031-11040.
8. Yoon, S.; Kim, J.; Lim, J.-H.; Yoo, B. Cobalt iron-phosphorus synthesized by electrodeposition as highly active and stable bifunctional catalyst for full water splitting. *Journal of The Electrochemical Society* **2018**, *165*, H271.
9. Pan, Y.; Sun, K.; Liu, S.; Cao, X.; Wu, K.; Cheong, W.-C.; Chen, Z.; Wang, Y.; Li, Y.; Liu, Y. Core–shell ZIF-8@ ZIF-67-derived CoP nanoparticle-embedded N-doped carbon nanotube hollow polyhedron for efficient overall water splitting. *Journal of the American Chemical Society* **2018**, *140*, 2610-2618.
10. Pei, Y.; Yang, Y.; Zhang, F.; Dong, P.; Baines, R.; Ge, Y.; Chu, H.; Ajayan, P.M.; Shen, J.; Ye, M. Controlled electrodeposition synthesis of Co–Ni–P film as a flexible and inexpensive electrode for efficient overall water splitting. *ACS applied materials & interfaces* **2017**, *9*, 31887-31896.
11. Chai, L.; Hu, Z.; Wang, X.; Xu, Y.; Zhang, L.; Li, T.T.; Hu, Y.; Qian, J.; Huang, S. Stringing bimetallic metal ‐ organic framework ‐ derived cobalt phosphide composite for high ‐ efficiency overall water splitting. *Advanced Science* **2020**, *7*, 1903195.
12. Lian, Y.; Sun, H.; Wang, X.; Qi, P.; Mu, Q.; Chen, Y.; Ye, J.; Zhao, X.; Deng, Z.; Peng, Y. Carved nanoframes of cobalt–iron bimetal phosphide as a bifunctional electrocatalyst for efficient overall water splitting. *Chemical science* **2019**, *10*, 464-474.
13. Wang, J.; Yang, W.; Liu, J. CoP 2 nanoparticles on reduced graphene oxide sheets as a super-efficient bifunctional electrocatalyst for full water splitting. *Journal of Materials Chemistry A* **2016**, *4*, 4686-4690.
14. Shi, J.; Qiu, F.; Yuan, W.; Guo, M.; Lu, Z.-H. Nitrogen-doped carbon-decorated yolk-shell CoP@ FeCoP micro-polyhedra derived from MOF for efficient overall water splitting. *Chemical Engineering Journal* **2021**, *403*, 126312.
15. Xiu, L.; Wang, Z.; Yu, M.; Wu, X.; Qiu, J. Aggregation-resistant 3D MXene-based architecture as efficient bifunctional electrocatalyst for overall water splitting. *ACS nano* **2018**, *12*, 8017-8028.
16. Zhang, P.; Lu, X.F.; Nai, J.; Zang, S.Q.; Lou, X.W. Construction of hierarchical Co–Fe oxyphosphide microtubes for electrocatalytic overall water splitting. *Advanced Science* **2019**, *6*, 1900576.
17. Anjum, M.A.R.; Okyay, M.S.; Kim, M.; Lee, M.H.; Park, N.; Lee, J.S. Bifunctional sulfur-doped cobalt phosphide electrocatalyst outperforms all-noble-metal electrocatalysts in alkaline electrolyzer for overall water splitting. *Nano Energy* **2018**, *53*, 286-295.
18. Yan, Y.; Xia, B.Y.; Ge, X.; Liu, Z.; Fisher, A.; Wang, X. A flexible electrode based on iron phosphide nanotubes for overall water splitting. *Chem.-Eur. J* **2015**, *21*, 18062-18067.
19. Jiao, L.; Zhou, Y.-X.; Jiang, H.-L. Metal–organic framework-based CoP/reduced graphene oxide: high-performance bifunctional electrocatalyst for overall water splitting. *Chemical Science* **2016**, *7*, 1690-1695.
20. Xue, Z.-H.; Su, H.; Yu, Q.-Y.; Zhang, B.; Wang, H.-H.; Li, X.-H.; Chen, J.-S. Janus Co/CoP Nanoparticles as Efficient Mott–Schottky Electrocatalysts for Overall Water Splitting in Wide pH Range. *Advanced Energy Materials* **2017**, *7*, 1602355, doi:https://doi.org/10.1002/aenm.201602355.
21. Anjum, M.A.R.; Bhatt, M.D.; Lee, M.H.; Lee, J.S. Sulfur-doped dicobalt phosphide outperforming precious metals as a bifunctional electrocatalyst for alkaline water electrolysis. *Chemistry of Materials* **2018**, *30*, 8861-8870.
22. Yang, Y.; Lin, Z.; Gao, S.; Su, J.; Lun, Z.; Xia, G.; Chen, J.; Zhang, R.; Chen, Q. Tuning electronic structures of nonprecious ternary alloys encapsulated in graphene layers for optimizing overall water splitting activity. *Acs Catalysis* **2017**, *7*, 469-479.
